# Correlation of Influenza B Haemagglutination Inhibiton, Single-Radial Haemolysis and Pseudotype-Based Microneutralisation Assays for Immunogenicity Testing of Seasonal Vaccines

**DOI:** 10.3390/vaccines9020100

**Published:** 2021-01-28

**Authors:** George W. Carnell, Claudia M. Trombetta, Francesca Ferrara, Emanuele Montomoli, Nigel J. Temperton

**Affiliations:** 1 Viral Pseudotype Unit, University of Kent and Greenwich, Chatham Maritime ME4 4TB, UK; gwc26@cam.ac.uk (G.W.C.); francesca.ferrara@stjude.org (F.F.); 2 Department of Molecular and Developmental Medicine, University of Siena, 53100 Siena, Italy; trombetta@unisi.it (C.M.T.); or emanuele.montomoli@vismederi.com (E.M.); 3 VisMederi srl, 53100 Siena, Italy

**Keywords:** influenza B viruses, serology

## Abstract

Influenza B is responsible for a significant proportion of the global morbidity, mortality and economic loss caused by influenza-related disease. Two antigenically distinct lineages co-circulate worldwide, often resulting in mismatches in vaccine coverage when vaccine predictions fail. There are currently operational issues with gold standard serological assays for influenza B, such as lack of sensitivity and requirement for specific antigen treatment. This study encompasses the gold standard assays with the more recent Pseudotype-based Microneutralisation assay in order to study comparative serological outcomes. Haemagglutination Inhibition, Single Radial Haemolysis and Pseudotype-based Microneutralisation correlated strongly for strains in the Yamagata lineage; however, it correlated with neither gold standard assays for the Victoria lineage.

## 1. Introduction

Influenza is a respiratory syndrome caused by three of six genera in the Orthomyxoviridae family, influenza A, B and C. A fourth genus (Influenza D) has also been characterised [1]. Influenza A virus is the most widespread; its various subtypes are classified according to their antigenically variable surface glycoproteins: haemagglutinin (HA, H1-H18) and neuraminidase (NA, N1-N11).

Influenza B viruses comprise two co-circulating, antigenically distinct lineages that diverged from their progenitor, strain B/Hong Kong/8/1973, into the “Yamagata-like” (B/Yamagata/16/1988 type) and “Victoria-like” (B/Victoria/2/1987 type) lineages [2]. This human virus causes a significant proportion (20–30%) of global morbidity associated with influenza virus disease due to its global distribution and unpredictable switches in the predominating lineage circulating [3,4,5]. The WHO vaccine recommendations include an up-to-date strain from both lineages for quadrivalent vaccines, but only one for trivalent vaccines. Should the circulating lineage not match the predicted lineage, there is an inevitable drop in coverage against influenza B-linked disease [3,6,7,8]. In the United States, multiple quadrivalent vaccines have been approved and are in use [9].

### 1.1. Serological Assays for Influenza B

Single Radial Immuno-Diffusion (SRID) has been one of the mainstays for the identification and characterisation of inactivated influenza vaccines, correlating with immunogenicity and clinical benefit/protection [10,11,12,13,14,15,16,17]. Haemagglutination Inhibition (HI) has been used for many decades as the tool used for the detection of influenza antibodies [18]. These assays, in combination with ELISA [19] and Single Radial Haemolysis (SRH) [20] comprise the gold standard assays for detection of influenza virus targeting antibodies and are generally applicable to the B type. However, more recent work has highlighted distinct shortcomings of the traditional assays, focusing research on the development of novel assays utilising various different technologies [21,22,23,24,25,26,27,28,29,30]. Ether treatment has been employed for influenza B viruses, prior to HI experiments, raising the efficacy of the HI test to that of the Complement Fixation (CF) test [31,32,33]. This technique was developed due to the lack of reactivity of certain strains of influenza, as well as the B type. Alongside the adaptation of SRID to influenza B resulting in SRH, historical use of ether treatment was found to increase sensitivity but reduces the specificity of HI during assay of serum samples against influenza B [20,31,32,34,35,36,37]

### 1.2. Monoclonal Antibodies

Monoclonal antibodies (mAbs) are increasingly being used in influenza research, whether for development of standards to complement or validate assays, or more directly to evaluate Haemagglutinin (HA) epitopes targeted through vaccination, or as potential therapeutics, as seen in the recent Ebola outbreak [38,39]. The mAbs employed in this study were developed as an antibody-based alternative for influenza B identity (Yamagata or Victoria lineages) and potency assays [40].

### 1.3. Study Aims

This study’s goal is determining the correlation of the gold standard assays HI and SRH with the recently adapted influenza B Pseudotype-based Microneutralisation assay (pMN), as HI detects primarily receptor binding site (RBS) proximal antibodies while SRH detects IgG1, IgG3 and IgM class antibodies that are compatible with the complement cascade. pMN detects HA-neutralising antibodies directed against both the globular head and the stalk [41]. As with SRH, HI has been correlated with protection against influenza, with titres at or above 40 linked to 50% or greater protection from infection in adults [12,42,43,44]. Linking either of these assays with pMN, despite the detection of different types of antibody, would allow more confidence to be attributed to the results generated using this assay, despite it actually detecting neutralisation of the function of the influenza HA glycoprotein. Correlation data between assays is important for in-depth interpretation of immunogenicity data, especially when correlation is determined with an assay that has been used for in vivo or challenge studies, establishing a correlate of protection. By inter-comparison of assays utilising different approaches, the scientific community can make more informed decisions on the future direction of vaccine design and testing. To date, no study of this type has been performed using influenza B, with efforts focusing more on the predominant A type [45,46]. As a major contributor to morbidity and mortality, it is essential to interrogate the correlations and relationships between data produced for influenza B using a range of assays. This is especially important as this type is lacking a definitive reservoir and circulates yearly as part of two lineages. In this study, influenza B lentiviral pseudotypes (PV) will be interrogated using a defined set of mAbs and a panel of sera, to put this resource to use in the correlation of pMN, SRH and HI.

## 2. Materials and Methods

### 2.1. Plasmids

Expression plasmid phCMV1 bearing the HA gene for B/Hong Kong/8/1973, B/Yamagata/16/1988 and B/Florida/4/2006 were obtained from Dr Davide Corti, Institute for Research in Biomedicine, Bellinzona, Switzerland. B/Victoria/60/2008, B/Brisbane/60/2008 and B/Bangladesh/3333/2007 HA genes were subcloned into plasmid pI.18. Plasmids p8.91 and pCSFLW were obtained from Prof. Greg Towers, University College, London, and originate from gene therapy applications [47,48]. To achieve maturation of HA gene products, protease-encoding plasmids were used [49,50,51,52], as detailed in Table 1 alongside the quantity transfected per well of a 6-well plate. Information regarding the strains of influenza B used can also be found in Table 1.

### 2.2. Serum Samples

One serum set was used per lineage of influenza B, consisting of samples taken pre and post seasonal vaccination. Paired low and high responders (based on HI results) were chosen. In total, 41 pairs of sera were assayed against B/Brisbane/60/2008 and 43 pairs against B/Florida/04/2006. Anonymised serum samples were obtained from Italian subjects and in compliance with Italian ethics law.

### 2.3. Pseudotype Production

Lentiviral pseudotypes were produced by transient transfection of HEK293T/17 cells with lentiviral packaging construct plasmids p8.91 [48] and pCSFLW [47] alongside an influenza B glycoprotein expression plasmid and corresponding protease expression plasmid as described in Section 2.1. Cells were transfected with 500 ng HA expression plasmid and corresponding protease listed in Table 1, alongside 500 ng p8.91 and 750 ng pCSFLW per well in a 6-well format. After 8 h, media was replaced and 1 unit of exogenous neuraminidase (*Clostridium perfringens*, Sigma, St. Louis, MO, USA) added per well to enable HA-pseudotype release. Cell culture supernatants were harvested 48 h post-transfection, filtered at 0.45 µm and titrated for transduction-based (firefly) luciferase activity on HEK293T/17 cells in 96-well format (Supplementary Figure S1).

### 2.4. Pseudotype-Based Microneutralisation Assay

Serial (1:2) dilutions of serum were performed in 96-well format in a total volume of 50 µL, and 50 µL of lentiviral pseudotype added to give a total relative luminescence (RLU) output of 1 × 10^6^ RLU per well. Serum and virus were incubated for 1 h at 37 °C, 5% CO_2_ in a humidified incubator, then 1.5 × 10^4^ HEK293T/17 cells were added per well. Plates were then incubated at 37 °C, 5% CO_2_ in a humidified incubator for 48 h, whereupon 50 µL of Bright-Glo (Promega) reagent was added per well and luminescence read after a 5 min incubation period. Data points were normalised to 100% and 0% neutralisation plate controls, and non-linear regressional analysis was performed to obtain neutralisation curves and IC_50_ and IC_90_ values. R^2^ values of 0.8 or more were used as a cut-off for confidence in the titration of antibody response, and any samples below this were discarded.

### 2.5. Haemagglutination Inhibition Assay

The influenza viruses used in the HI were B/Brisbane/06/2008 (15/146) and B/Florida/04/2006 (08/138), obtained from the National Institute for Biological Standards and Control (NIBSC), United Kingdom. Serum samples were pre-treated with receptor destroying enzyme (RDE) from *Vibrio cholerae* (Sigma Aldrich) at 1:5 ratio for 18 h at 37 °C in a water bath and then heat-inactivated for 1 h at 56 °C in a water bath with 8% sodium citrate at a 1:4 ratio. Turkey erythrocytes (Emozoo S.N.C, Casole d’Elsa, Italy) were centrifuged twice, washed with a saline solution (0.9%) and adjusted to a final dilution of 0.35%. RDE-treated serum samples were diluted two-fold with saline solution (0.9%) in a 96-well plate, starting from an initial dilution of 1:10. 25 µL of influenza virus was added to each well, and the mixture was incubated at room temperature for 1 h. At the end of the incubation, erythrocytes were added and the plates were evaluated for the inhibition of agglutination after 1 h at room temperature.

The antibody titre was expressed as the reciprocal of the highest serum dilution showing complete inhibition of agglutination. Since the starting dilution was 1:10, the lower limit of detectable antibody titre was 10. When the titre was below the detectable threshold, the results were conventionally expressed as 5 for calculation purposes, half the lowest detection threshold. Geometric mean titers were calculated from experimental repeats.

### 2.6. Single Radial Haemolysis Assay

The influenza viruses B/Brisbane/06/2008 (15/146) and B/Florida/04/2006 (08/138) were obtained from the NIBSC. Serum samples were heat-inactivated at 56 °C for 30 min in a water bath. Turkey erythrocytes were centrifuged twice and washed with phosphate-buffered saline (PBS). Diluted virus was added to the erythrocyte suspension at a concentration of 2000 haemagglutination units (HAU) per mL. The erythrocyte–virus suspension was incubated at 4 °C for 20 min, and subsequently, a solution of 2.5 mM Chromium Chloride (CrCl_3_) was added to the suspension, and it was incubated at room temperature for 10 min. The suspension was then carefully mixed once and then centrifuged. A stock solution consisting of 1.5% agarose in PBS containing 0.1% sodium azide and 0.5% low gelling agarose was prepared. This agarose stock solution was maintained at 45 °C in a water bath. The final suspension of erythrocytes, virus and guinea pig complement was vigorously shaken and evenly spread onto each plate. Plates were incubated at room temperature for 30 min and then stored at 4 °C for 30–90 min. Holes were introduced into each plate with a calibrated punch, and 6 µL of each serum sample was dispensed into each hole. The plates were stored in a humid box and incubated at 4 °C for 16–18 h in the dark. Subsequently, plates were incubated in a water bath at 37 °C for 90 min; diameters of the areas of haemolysis were then read in mm with a calibrating viewer [20,53].

### 2.7. mAbs and Controls

Influenza B mAbs were kindly provided by Dr Jerry P Weir, Division of Viral Products, Food and Drug Administration (FDA), USA [40]; see Table 2. Anti-B/Brisbane/60/2008 HA serum (11/136) was obtained from the NIBSC and employed against all strains of influenza B as a serum positive control. This antiserum had previously been tested in our laboratory, showing that it was capable of neutralising all available strains of influenza B to varying degrees, with the highest potency against the matched B/Brisbane/60/2008 strain [54].

### 2.8. Statistical Analysis

Mean endpoint antibody titres (or IC_50_ for pMN) for each serum sample were compared between assays. This comparison was carried out in GraphPad Prism where a two-tailed Pearson’s correlation was performed on pairs of data sets that were plotted against each other. A line of best fit was used in order to show the general trend of correlation between data sets. Analysis was performed between pre- (V1) and post-vaccination (V2) sera, and fold change from V1 to V2. pMN data consisted of both IC_50_ and IC_90_ values, and further analysis was carried out on transformed (log10) V2 data.

## 3. Results

### 3.1. Lineage Specific and Cross-Reacting mAbs Neutralise Influenza B PV

**Figure 1:**
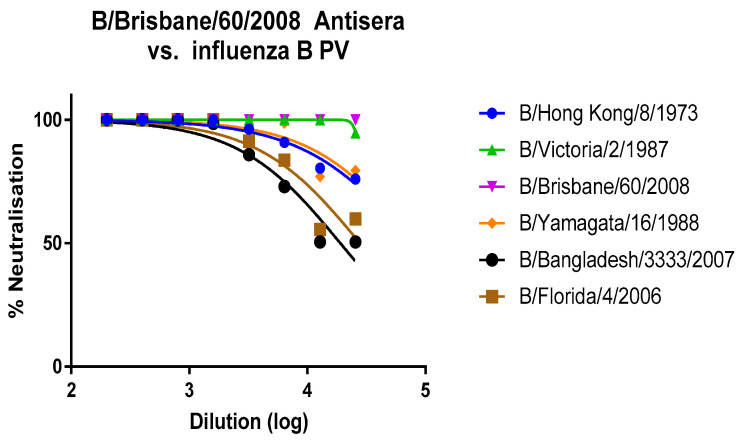
Anti-B/Brisbane/60/2008 HA serum (11/136, NIBSC) neutralises matched and related strains B/Brisbane/60/2008 and B/Victoria/2/1987 the strongest but also neutralises Yamagata lineage strains and the B/Hong Kong/7/1973 precursor to a lesser extent.

Lineage-specific mAbs neutralised all influenza B PV except B/Brisbane/60/2008, which was not susceptible in our experiments. This PV was unaffected by either the Victoria specific mAbs, or the 2F11 cross-lineage mAb. Despite this, anti-B/Brisbane/60/2008 HA serum showed the highest neutralisation against the matched B/Brisbane/60/2008 pseudotypes (IC_50_ > 256,000) (Figure 1). The 2F11 mAb neutralised Yamagata lineage strains the strongest (IC_50_: 2–4 ng/mL), followed by the pre-lineage strain B/Hong Kong/8/1973 (IC_50_: 8 ng/mL) and finally the Victoria strain B/Victoria/2/1987 (IC_50_: 160 ng/mL). Yamagata lineage-specific mAbs 3E8 and 1H4 were only effective on Yamagata lineage PVs, neutralising B/Bangladesh/3333/2007, the strongest (IC_50_: 3 and 4 ng/mL, respectively), followed by B/Florida/4/2006 (IC_50_: 21 and 3 ng/mL) and B/Yamagata (IC_50_: 34 and 6 ng/mL). Victoria-specific mAbs 8E12 and 5A1 neutralised B/Hong Kong the strongest (IC_50_: 2 and 4 ng/mL, respectively), followed by B/Victoria/2/1987 (IC_50_: 47 and 22). See Table 3 for a list of IC_50_ values.

Anti B/Brisbane/60/2008 polyclonal sheep serum was used as an anti-influenza B control in all of these experiments and for all strains of influenza B PV due to its ability to neutralise all strains tested in the pMN assay, regardless of lineage. This antiserum is most potent against the homologous strain B/Brisbane/60/2008 and the other Victoria lineage strain B/Victoria/2/1987 (Figure 4), and 100% neutralisation is seen from 1:200 up to the penultimate dilution point 1:128,000.

**Figure 2:**
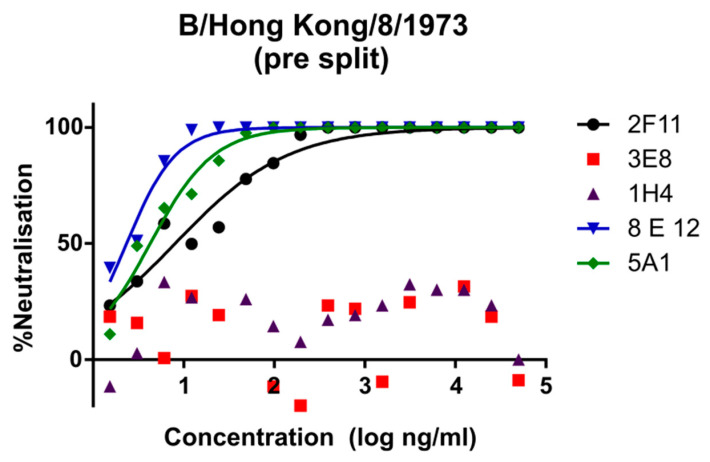
pMN neutralisation curves for FDA mAbs 2F11, 3E8, 1H4, 8E12 and 5A1 against PV bearing the HA glycoprotein from the influenza B pre-lineage split strain, B/Hong Kong/8/1973. This PV is neutralised strongly by Victoria-specific mAbs 5A1, 8E12 and cross-lineage mAb 2F11 but not Yamagata specific mAbs 3E8 or 1H4.

**Figure 3:**
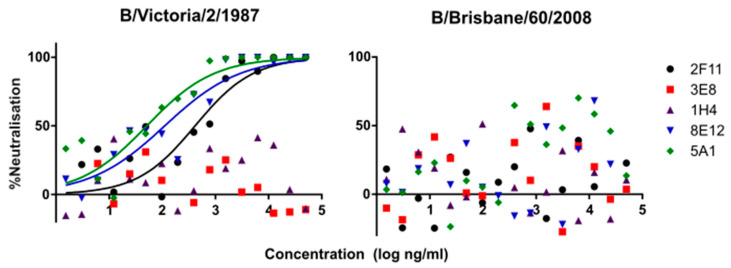
pMN neutralisation curves for FDA mAbs 2F11, 3E8, 1H4, 8E12 and 5A1 against PV bearing HA glycoproteins from Victoria lineage strains B/Victoria/2/1987 and B/Brisbane/60/2008. Victoria lineage PVs are not neutralised by Yamagata-specific mAbs 3E8 or 1H4. A/Victoria/2/1987 PV is neutralised by Victoria lineage-specific mAbs 5A1, 8E12 and cross-lineage mAb 2F11. B/Brisbane/60/2008 PV are not neutralised by any of the mAbs tested, including Victoria lineage and cross-lineage mAbs with a high degree of assay variability.

**Figure 4:**
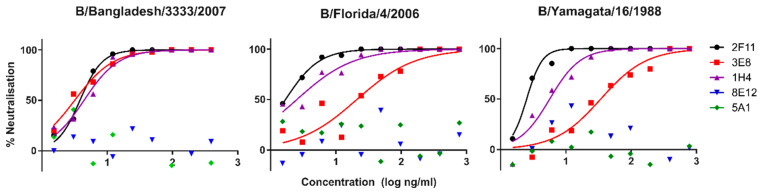
pMN neutralisation curves for FDA mAbs 2F11, 3E8, 1H4, 8E12 and 5A1 against PV bearing HA glycoproteins from Yamagata lineage strains B/Yamagata/16/1988, B/Florida/4/2006 and B/Bangladesh/3333/2007. Potent neutralisation by Yamagata specific mAbs 3E8 and 1H4, and by cross-lineage mAb 2F11. No neutralisation is observed for Victoria lineage-specific mAbs 8E12 and 5A1.

The data for the Victoria lineage viruses show reduced antibody potency, with IC_50_ values up to 20 times higher. Victoria epitope targeting mAbs neutralise B/Victoria as well as the Hong Kong precursor. Neutralisation curves for the above data are shown in Figure 2, Figure 3 and Figure 4.

### 3.2. Correlation of SRH, HI and pMN

**Figure 5:**
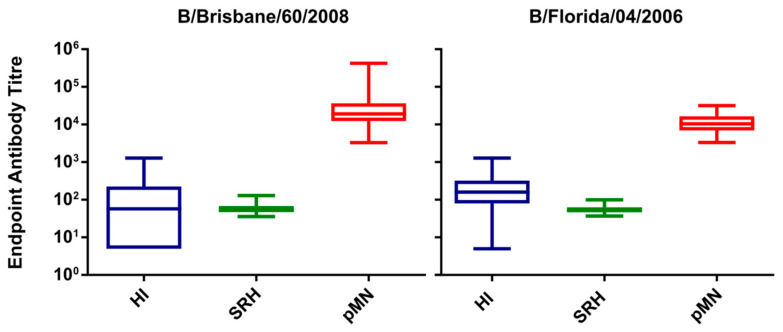
Haemagglutination Inhibition (HI), Single Radial Haemolysis (SRH) endpoint titres and pMN IC_50_ values for high responders (V2) against B/Brisbane/60/2008 and B/Florida/04/2006. Titres displayed show similar profiles for each strain tested, with a broader range of pMN IC_50_ values for B/Brisbane/60/2008.

SRH, HI and pMN data for strains B/Florida/04/2006 (Yamagata) and B/Brisbane/60/2008 (Victoria) were analysed to assess the correlation between each assay. Endpoint titres for high responders (V2) are displayed in Figure 5. The titre profiles of each serum set are very similar between the two viruses, despite each virus having its own unique set of sera. HI values range from 5 to 1280 for both B/Brisbane/60/2008 and B/Florida/04/2006, with arithmetic means at 156 and 270, respectively. SRH values range from 37 to 106 and 38 to 99 mm^2^ for B/Brisbane/60/2008 and B/Florida/04/2006, respectively. Arithmetic means were 58 and 57 mm^2^, respectively. pMN (IC_50_) values ranged from 4594 to 209,395 for B/Brisbane/60/2008 and 3349 to 31,954 for B/Florida/04/2006. Arithmetic mean pMN titre means were 41,613 and 11,740, respectively.

### 3.3. Correlation of Data: B/Brisbane/60/2008 IC_50_

**Figure 6:**
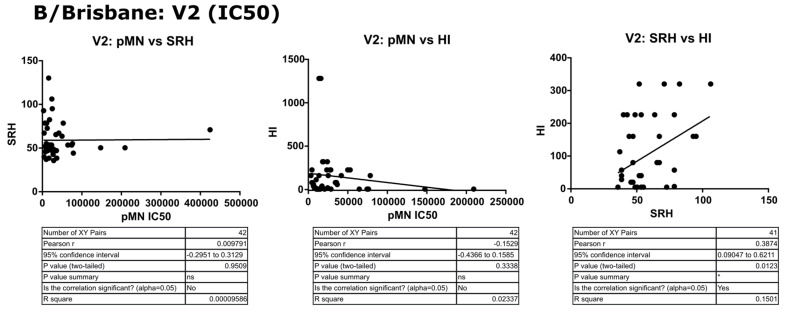
Correlation of SRH, HI and pMN (IC_50_) mean V2 values assayed against B/Brisbane/60/2008 PV. Pearson’s two-tailed analysis performed using GraphPad Prism. No correlation was observed between IC_50_ antibody titres and SRH or HI (Pearsons r = 0.009 and −0.152, respectively). SRH and HI values were correlated (Pearsons r = 0.38). *p* > 0.05 = ns, *p* ≤ 0.05 = *.

**Figure 7:**
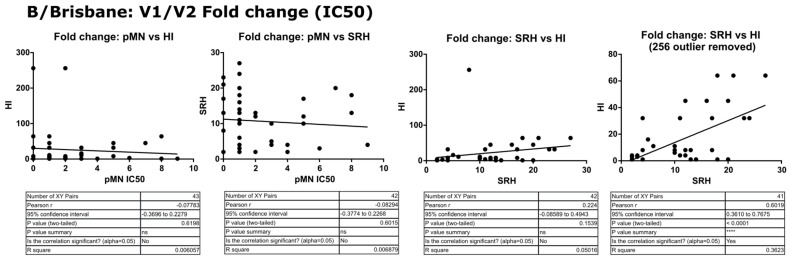
Correlation of SRH, HI and pMN (IC_50_) V1 to V2 fold-change values assayed against B/Brisbane/60/2008 PV. Pearson’s two-tailed analysis performed using GraphPad Prism. No correlation was observed between IC_50_ antibody titres and HI or SRH (Pearsons r = −0.07 and −0.08, respectively). Weak correlation was observed between SRH and HI (Pearsons r = 0.22), which was improved after removal of outlying HI value (Pearsons r = 0.60). *p* > 0.05 = ns, *p* ≤ 0.0001 = ****.

Two different sets of values for each assay were correlated, V2 data (post-vaccination high responders) and fold change between V1 and V2. For B/Brisbane/60/2008, no correlation was observed between pMN and the other assays, with Pearson’s r values ranging from −0.07 to −0.08 (*p* = 0.13 to 0.9). SRH and HI correlated when fold changes were compared (r = 0.224, *p* = 0.1539) and increased after the removal of two outliers (r = 0.60, *p* ≥ 0.0001). V1 results weakly correlated (r = 0.32, *p* = 0.0375) for SRH and HI, and more so for V2 results (r = 0.39, *p* = 0.0123). See Figure 6 and Figure 7 for correlation graphs and data.

### 3.4. Correlation of Data: B/Brisbane IC_90_ and Transformed Data

**Figure 8:**
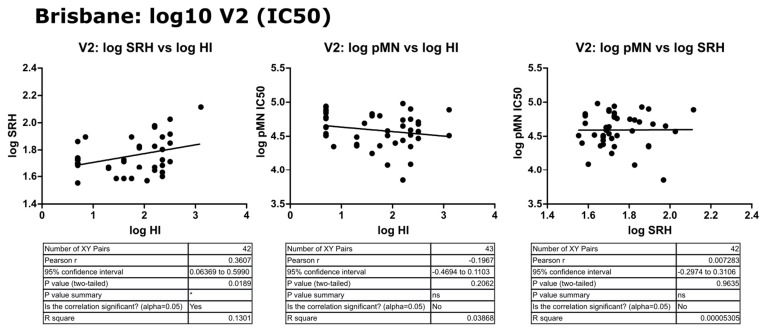
Correlation of transformed (log10) SRH, HI and pMN (IC_50_) mean V2 values assayed against B/Brisbane/60/2008 PV. Pearson’s two-tailed analysis performed using GraphPad Prism. Weak correlation was observed between log SRH and HI values (Pearsons r = 0.36). No correlation was observed between log IC_50_ antibody titres and log HI or SRH values (Pearsons r = −0.196 and 0.007, respectively). *p* > 0.05 = ns, *p* ≤ 0.05 = *.

**Figure 9:**
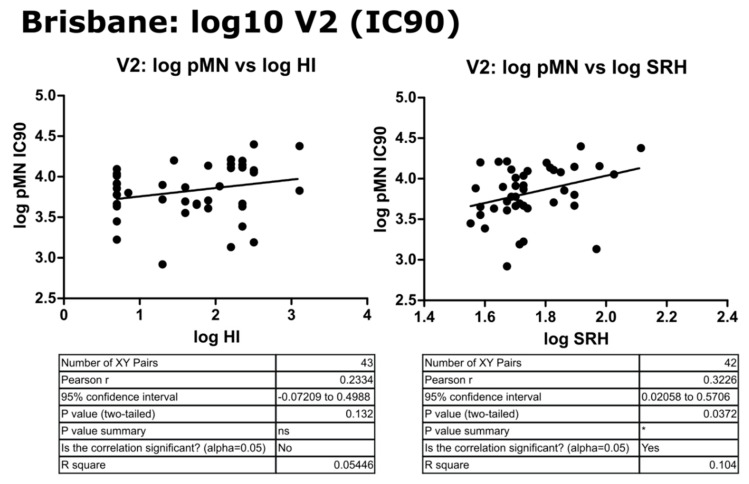
Correlation of transformed (log10) data for pMN (IC_90_) with HI and SRH using mean V2 values for sera assayed against B/Brisbane/60/2008 PV. Pearson’s two-tailed analysis performed using GraphPad Prism. Weak correlation was observed between log IC_90_ antibody titres and log HI or SRH values (Pearsons r = 0.23 and 0.32, respectively). *p* > 0.05 = ns, *p* ≤ 0.05 = *.

**Figure 10:**
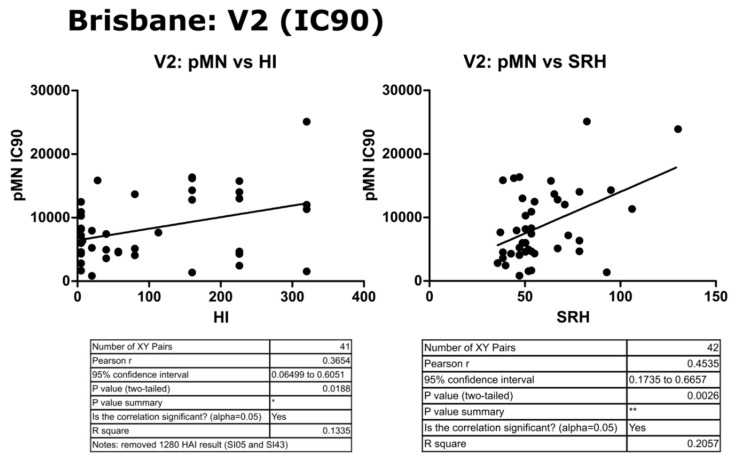
Correlation of pMN (IC_90_) with HI and SRH using mean V2 values for sera assayed against B/Brisbane/60/2008 PV. Pearson’s two-tailed analysis performed using prism Graph Pad. Weak correlation was observed between IC_90_ antibody titres and HI (Pearsons r = 0.36). IC_90_ values correlated well with SRH values (Pearsons r = 0.45). *p* ≤ 0.05 = *, *p* ≤ 0.01 = **.

Upon transformation of the V2 data to a log10 scale, correlation was still not observed between pMN and SRH or HI despite normalization of scales, with a weak correlation between the latter two (r = 0.35, *p* = 0.0189). pMN IC_90_ data correlated weakly with HI and SRH (r = 0.37 and 0.45, *p* = 0.00188 and 0.0026, respectively). The correlation seen was reduced by a transformation of IC_90_ data and comparison on a log scale (r = 0.23 and 0.32, *p* = 0.132 and 0.0372, respectively) (see Figure 8, Figure 9 and Figure 10).

### 3.5. Correlation of Data: B/Florida/4/2006 IC_50_

**Figure 11:**
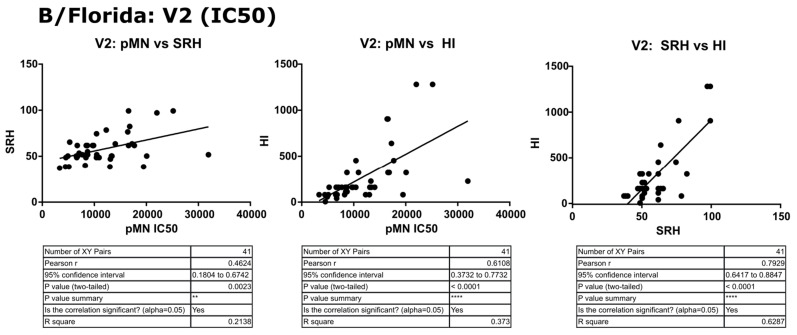
Correlation of SRH, HI and pMN (IC_50_) mean V2 values assayed against B/Florida/4/2006 PV. Pearson’s two-tailed analysis performed using GraphPad Prism. Weak correlation was observed between IC_50_ antibody titres and SRH (Pearsons r = 0.46). Strong correlation was observed between IC_50_ antibody titres and HI (Pearsons r = 0.61), as well as between SRH and HI (Pearsons r = 0.79) for the V2 data tested. *p* ≤ 0.01 = **, *p* ≤ 0.0001 = ****.

**Figure 12:**
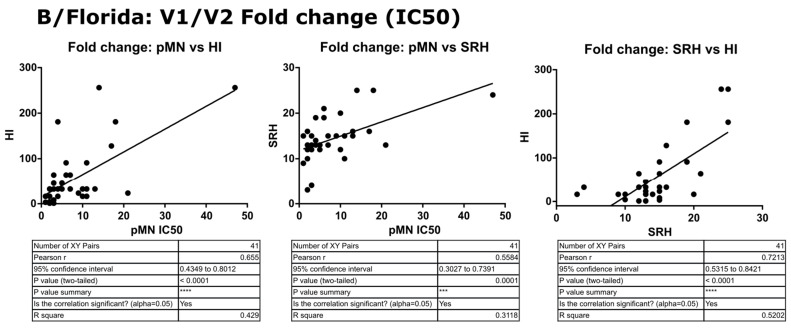
Correlation of SRH, HI and pMN (IC_50_) V1 to V2 fold change values assayed against B/Florida/4/2006 PV. Pearson’s two-tailed analysis performed using GraphPad Prism. Strong correlation was seen between fold change in pMN and HI values between V1 and V2 antibody titres (IC_50_), Pearsons r = 0.65. pMN and SRH V1/V2 fold changes correlated well, Pearsons r = 0.55 and strong correlation was observed for V1/V2 fold change for antibody titres of SRH and HI (Pearsons r = 0.72). *p* ≤ 0.001 = ***, *p* ≤ 0.0001 = ****.

In contrast to the results for B/Brisbane/60/2008, pMN data correlated well with SRH and HI, and V2 SRH and HI correlated strongly (r = 0.79, *p* ≤ 0.0001) (see Figure 11). For the fold change data, pMN correlated with HI and SRH (r = 0.66 and 0.56, *p* ≤ 0.0001). SRH and HI correlated strongly (r = 0.72, *p* ≤ 0.0001) (see Figure 12). For V2 data, pMN correlated with SRH (r = 0.46, *p* = 0.0023) and HI (r = 0.61, *p* ≥ 0.0001).

### 3.6. Correlation of Data: B/Florida/4/2006 IC_90_ and Transformed Data

**Figure 13:**
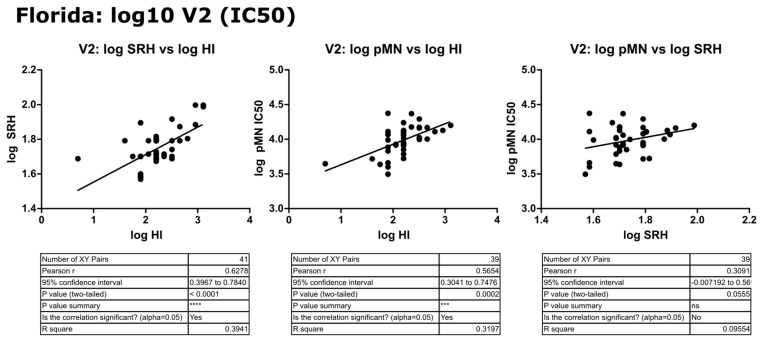
Correlation of transformed (log10) SRH, HI and pMN (IC_50_) mean V2 values assayed against B/Florida/4/2006 PV. Pearson’s two-tailed analysis performed using GraphPad Prism. Strong correlation is observed between log SRH and HI (Pearsons r = 0.62. log pMN and log HI correlate well (Pearsons r = 0.56), and weak correlation was seen between log pMN and log SRH (Pearsons r = 0.30). *p* > 0.05 = ns, *p* ≤ 0.001 = ***, *p* ≤ 0.0001 = ****.

**Figure 14:**
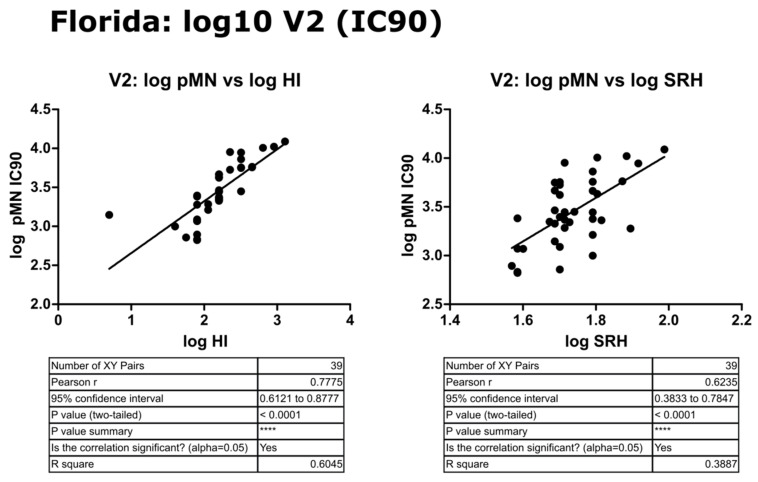
Correlation of transformed (log10) data for pMN (IC_90_) with SRH and HI mean V2 values, assayed against B/Florida/4/2006 PV. Pearson’s two-tailed analysis performed using GraphPad Prism. Strong correlation was observed between log IC_90_ antibody titres and log HI or SRH values, with Pearsons r values of 0.77 and 0.62, respectively. *p* ≤ 0.0001 = ****.

**Figure 15:**
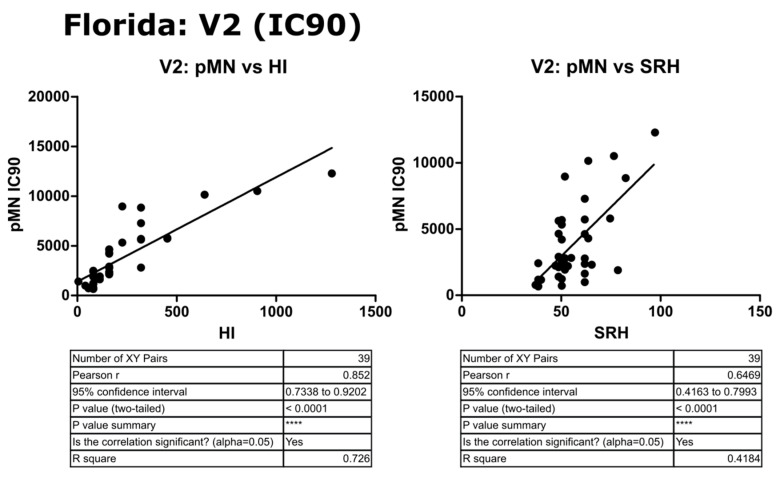
Correlation between pMN (IC_90_) with SRH and HI mean V2 values, assayed against B/Florida/4/2006 PV. Pearson’s two-tailed analysis performed using GraphPad prism. Strong correlation was observed between IC_90_ antibody titres and HI or SRH, with Pearsons r values of 0.85 and 0.64, respectively. *p* ≤ 0.0001 = ****.

Transformation to a log10 scale caused a slight reduction in the significance attributed to the correlation between pMN and SRH or HI (r = 0.57 or 0.30, *p* = 0.0002 or 0.0555), but correlation remained strong between all three assays. IC_90_ data correlated very well between pMN and SRH or HI (r = 0.85 and 0.65, *p* ≤ 0.0001) and this was maintained when data were transformed to the log10 scale (r = 0.78 and 0.62, *p* ≤ 0.0001) (see Figure 13, Figure 14 and Figure 15).

## 4. Discussion

Neutralisation of influenza B bearing PV with influenza B-specific mAbs was consistent, with the exception of B/Brisbane/60/2008 PV. Victoria-lineage-specific mAbs neutralised the type B/Victoria/2/1987 PV, while Yamagata-lineage-specific mAbs neutralised Yamagata lineage strains (Figure 1, Figure 2 and Figure 3). The cross-lineage-specific mAb 2F11 neutralised both lineages as well as the pre-lineage split strain B/Hong Kong/8/1973. Discordant correlation was observed between Victoria and Yamagata lineages, with the former correlating well between pMN, SRH and HI and the latter only correlating, weakly, between SRH and HI.

### 4.1. Neutralisation by Influenza B mAbs

While the majority of influenza B mAbs generated by the FDA neutralised the expected strains in the pMN assay, neutralisation was not seen for B/Brisbane/60/2008 by Victoria-specific mAbs, in contrast to data supporting the characterisation of these mAbs [40].

One possible explanation for this is N-linked glycosylation masking epitopes on the HA surface. The presence or absence of a glycosylated residue would dramatically affect results when neutralising with a mAb targetting one specific epitope. In this study, a WT B/Brisbane/60/2008 gene was used to produce PV for use in pMN, while B/Victoria/2/1987 was human-codon-optimised. HI and SRH made use of inactivated antigen produced in bacteria, whereas Verma and colleagues used a combination of ELISA using inactivated antigen and PV assays, with the latter using an egg-adapted strain of B/Brisbane/60/2008. Studies on egg-adaptation of influenza B viruses have characterised mutations within the 190 helix loop of the receptor-binding domain, which led to a significant change in antigenicity of the HA [55,56] due to the loss of an N-linked glycosylation site [57]. Despite the lack of neutralisation shown against B/Brisbane/60/2008 by the Victoria-specific mAbs in this study, polyclonal hyperimmune antisera produced against the same HA subtype was the most effective at neutralising it (Figure 4), suggesting that the HA itself is antigenically correct and that the problem lies in the specific epitopes targeted by 8E12 and 5A1. These mAbs were reported to bind to the amino acid residues 241 and 203, respectively, which are within or close to the 190 helix, which spans from residue 195 to 235 [40,58].

The fact that mAbs specific for the Victoria lineage also neutralised the pre-lineage split strain (Figure 1) is not surprising, as fewer structural differences have been reported between these than between the pre-lineage strain and the Yamagata lineage. The positions of escape mutations generated against the Victoria lineage-specific mAbs 8E12 and 5A1 presented in their characterisation were P241Q and K203R, respectively [40]. These are present within the 190 helix (RBD) of Victoria strains, which is very similar in amino acid composition to the 190 helix of the pre lineage split strain B/Hong Kong/8/1973 [58].

### 4.2. Correlation between SRH and HI

Overall, SRH and HI correlated strongly across both lineages, except for in V1 samples, which were predictably very low due to the lower sensitivity of these assays. As most of the V1 samples were negative in terms of HI and SRH data, they were clustered around the respective titres of 5 and 4 mm^2^, which led to a lack of correlation through analysis. Fold change and V2 samples correlated for both Victoria and Yamagata lineages, indicating that increases in influenza B HA-specific antibodies are detected by both assays.

### 4.3. Correlation between pMN and SRH

pMN correlated strongly with SRH for the Yamagata lineage strain, with correlation observed for all data sets except for the negative or low titre samples that clustered to the lower detection limit of SRH at 4 mm^2^. This suggests that pMN would be preferable for the evaluation of low-response samples, as its sensitivity offers an advantage over SRH. For B/Brisbane/60/2008, however, the only correlation observed was between IC_90_ values (transformed and raw data). Despite this, Pearson’s r was still below 0.5 in each case.

### 4.4. Correlation between pMN and HI

As with pMN and SRH, a strong correlation is seen for the Yamagata strain (Figure 6, Figure 7, Figure 8, Figure 9 and Figure 10) but not the Victoria strain (Figure 8, Figure 9, Figure 10, Figure 11 and Figure 12); once again, B/Brisbane/60/2008 only poorly correlates when analysis is performed using IC_90_ values (Figure 10). B/Brisbane/60/2008 IC_50_ antibody titres do not correlate with SRH or HI when using either fold-change or V2 data, transformed or raw.

### 4.5. Limitations of This Study

Influenza B viruses have not undergone the ether treatment that could enhance their performance in the HI assay, despite reducing the assay specificity. The pseudotype-based assay represents a novel platform that is increasingly used by laboratories worldwide. However, each assay and antigen requires validation and optimization before this assay can compete with traditional HI or MN assays, which remain the gold standard for immunological outputs. In addition, as this was a single-cycle assay, the effect of neutralising or interfering antibodies on viral egress was not measured, contrary to live virus assays such as MN or PRNT, where an effect can be measured on viral egress. In our hands, the B/Brisbane/60/2008 PV did not perform as expected against characterised monoclonal antibodies, despite being strongly neutralised by polyclonal serum raised against the same antigen. This was a small panel of mAbs that would benefit from being expanded and evaluated on B/Brisbane/60/2008.

## 5. Conclusions

The results presented in this study, while discordant in regards to B/Brisbane/60/2008 PV, still represent the first comprehensive study correlating HI, SRH and pMN assays for influenza B serology. Lentiviral pseudotypes are becoming increasingly popular as a surrogate for live virus neutralisation assays—especially in current R&D using containment level 3 or 4 pathogens such as SARS-CoV-2 and Ebolavirus. The exquisite sensitivity of this assay allows the differentiation of serum samples that would otherwise be categorized as negative in the gold standard assays. This sensitivity can be a double-edged sword, as serum samples and reference antisera against the influenza B lentiviral pseudotypes reported in this study were strongly neutralising, leading to exceptionally high IC_50_ titers that may have been an overestimation of the serum potency. The nature of each assay described in this study is unique and detects antibodies that may interfere at different points in the virus life cycle. Neutralising antibodies are among the most important and often the goal for universal vaccine approaches—but are not explicitly detected by older assays including SRH and ELISA.

Many efforts have been made to standardise assays between platforms and laboratories to facilitate the comparison of different projects and data sets, such as the efforts by the Consortium for the Standardization of Influenza Seroepidemiology (CONSISE, https://consise.tghn.org/) and the establishment of reference standards and calibrants by the National Institute for Biological Standards and Control (NIBSC, nibsc.org). Until each pseudotyped virus has been fully validated and compared to traditional microneutralisation in a parallel and controlled setting, the gold standard assays will remain preferable for laboratories that are able to perform live virus assays.

## Figures and Tables

**Table 1:** Influenza B strains, accession numbers, lineage, protease type and quantity required for production in 6-well format are displayed. Human Airway Trypsin (HAT) and Transmembrane protease, serine 4 (TMPRSS4) expression plasmids were co-transfected with other plasmids to allow maturation of HA0 to HA1/2 inside producer cells.

Strain	Accession	Lineage	Protease	ng per 6-Well
B/Hong Kong/8/1973	K00425	Pre-split	HAT	125
B/Victoria/2/1987	FJ766840	Victoria	HAT	125
B/Brisbane/60/2008	KX058884	Victoria	TMPRSS4	125
B/Yamagata/16/1988	CY018765	Yamagata	HAT	125
B/Florida/4/2006	EU515992	Yamagata	HAT	125
B/Bangladesh/3333/2007	CY115255	Yamagata	HAT	250

**Table 2:** Influenza B mAbs. Five mAbs used, two specific for each lineage of influenza B and one binding to an epitope conserved between both lineages (cross).

mAb	2F11	3E8	1H4	8E12	5A1
Target lineage	Cross	Yamagata	Yamagata	Victoria	Victoria

**Table 3:** IC_50_ values in ng/mL for the neutralisation of 6 strains of influenza B HA-PV by mAbs. Pre-lineage strain, Yamagata and Victoria lineages highlighted in grey, green and blue, respectively. With the exception of B/Brisbane/60/2008, the cross lineage mAb 2F11 acts as expected, neutralising all influenza B PV. Yamagata specific mAbs neutralise PV bearing HAs from Yamagata lineage strains, while Victoria-specific mAbs neutralise PV bearing HAs from Victoria lineage strains with the exception of B/Brisbane/60/2008. The pre-lineage spli t strain B/Hong Kong/8/1973 is neutralised in a Victoria-like manner by 2F11 and Victoria lineage-specific mAbs 8E12 and 5A1.

mAb	Target	B/Hong Kong/8/1973	B/Florida/4/2006	B/Bangladesh/3333/2007	B/Victoria/2/1987	B/Brisbane/60/2008
2F11	Cross	8	3	2	4	-
3E8	Yamagata	-	34	21	3	-
1H4	Yamagata	-	6	3	4	-
8E12	Victoria	2	-	-	-	-
5A1	VIctoria	4	-	-	-	-

## Data Availability

The data presented in this study are available on request from the corresponding author.

## Supplementary Materials

The following are available online at https://www.mdpi.com/2076-393X/9/2/100/s1, Figure S1: Titration of luciferase pseudotypes bearing the B/Brisbane/60/2008 haemagglutinin on HEK293T/17 cells. Quality control and production optimisation can be found as described previously.
