# Supplementary material for: Correlation of Influenza B Haemagglutination Inhibiton, Single-Radial Haemolysis and Pseudotype-Based Microneutralisation Assays for Immunogenicity Testing of Seasonal Vaccines
Source: Vaccines (Basel). 2021 Jan 28;9(2):100. doi: 10.3390/vaccines9020100 (PMC7911544; doi:10.3390/vaccines9020100)
Supplement: Supplementary file 1 [file vaccines-09-00100-s001.pdf]

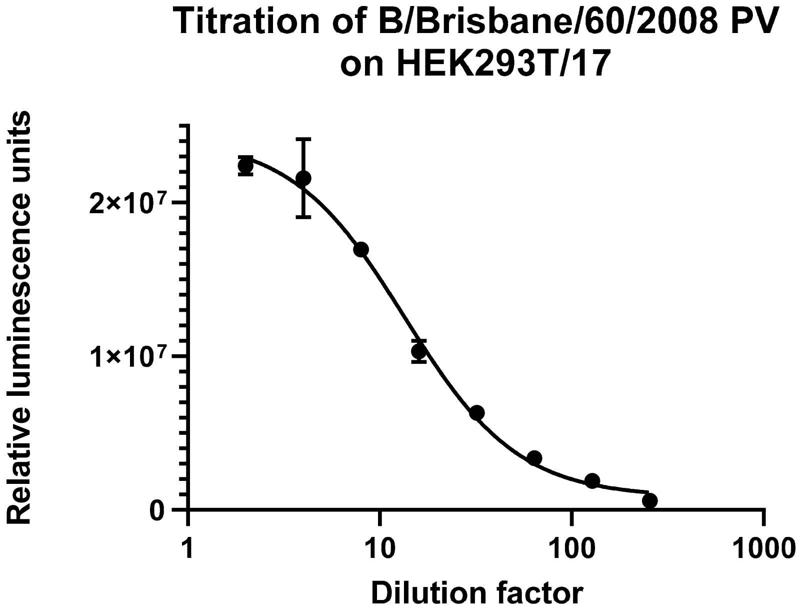

Supplementary figure 1. Titration of luciferase pseudotypes bearing the B/Brisbane/60/2008 haemagglutinin on HEK293T/17 cells. Quality control and production optimisation can be found as described previously (Ferrara et al. 2018).

Supplementary references:

Ferrara, F., Carnell, G., Kinsley, R., Böttcher-Friebertshäuser, E., Pöhlmann, S., Scott, S., et al. (2018). Development and use of lentiviral vectors pseudotyped with influenza B haemagglutinins: Application to vaccine immunogenicity, mAb potency and sero-surveillance studies. *bioRxiv*. https://doi.org/10.1101/492785
